# The Behavior of Patients with Obsessive-Compulsive Disorder in Dental Clinics

**DOI:** 10.1155/2021/5561690

**Published:** 2021-08-30

**Authors:** Hala M. Elkamash, Hatem M. Abuohashish

**Affiliations:** ^1^Egypt Ministry of Health and Population, Alexandria 21500, Egypt; ^2^Department of Biomedical Dental Sciences, College of Dentistry, Imam Abdulrahman Bin Faisal University, Dammam 31441, Saudi Arabia

## Abstract

**Objectives:**

This review documents published obsessive-compulsive disorder (OCD) cases with dental and oral conditions with potential impact on the dental procedure. The research question was, what are the psychiatric and behavioral features of people with OCD that might affect dental sessions?

**Methods:**

This review followed the PRISMA guidelines (PROSPERO registration No. CRD42020212371). Six databases (PubMed, Scopus, Web of Science, LILACS, Cochrane Library, and PsycINFO) were screened for published clinical studies that report dental patients with obsessions or compulsions behaviors as identified by National Institute of Mental Health (NIMH). Inclusion of the studies was performed according to the eligibility criteria. The quality evaluation was carried out using the Joanna Briggs Institute's (JBI) Critical Appraisal Checklist. The results were qualitatively assessed for synthesis.

**Results:**

After elimination of duplication, 530 articles were screened, and 35 articles were evaluated for eligibility. 17 studies met the inclusion criteria (8 case reports, 5 cross-sectional studies, 1 longitudinal cohort study, and 3 case-control studies) and were included in the review. All case reports demonstrated symptoms of obsessions or compulsions such as fear of germs and contamination, aggressive thoughts, having things symmetric in perfect order, excessive cleaning or handwashing, repeatedly checking things, and compulsive counting. OCD-related behavior was assessed in the included clinical investigations using standardized protocols such as Florida Obsessive-Compulsive Inventory, Symptom Checklist-90-Revised, 4-item Corah Dental Anxiety Scale, Diagnostic and Statistical Manual of Mental Disorders, and the Crown Crisp Experimental Index. Quality assessment of the 17 included articles revealed 14 articles with low risk of bias and 3 articles with moderate risk of bias.

**Conclusion:**

The reported OCD symptoms may implement psychological difficulties during dental procedures without affecting the outcome. Although there was no contraindication for planning or performing dental treatments for a patient with OCD, dental-related procedures and protocols might be modified for successful dental appointments.

## 1. Introduction

Obsessive-compulsive disorder (OCD) is a severe psychological disorder, with global prevalence of 2-3% [1]. The major characteristics of OCD include obsessional debilitating inner thoughts associated with repetitive behaviors [1]. OCD can be presented in four types of obsessive symptoms as identified by the National Institute of Mental Health (NIMH) including (1) fear of infection, germs diseases, and contamination; (2) aggressive thoughts against self or others; (3) impious thoughts; and (4) worries about symmetry and perfectionism [2]. According to these intrusive thoughts, obsessive patients feel the urge toward some behaviors as frequent washing and cleaning, checking things repetitively, and compulsive counting. OCD always shows two modes of onset. By approaching 11 years of age, the first peak starts and then in the early adulthood the second peak begins. Almost by 10 years of age, symptoms appear in around 20% of the affected population [3].

Several psychological symptoms might hinder the success of dental procedures and can give the dentist hard time during treatment periods. Due to fear of infection, patients with OCD may be distrusted about the disinfection and cleanliness of the materials, tools, table, and dental chair, which may irritate the dentist. Therefore, taking a complete medical history, including psychiatric, along with consulting the patient's psychiatric physician is a vital step to avoid dental problems or oral conditions. Moreover, dentists should be aware of OCD diagnostic criteria as they might be the first ones to suspect that their patients may be diagnosed with OCD by correlating patient's behavior with OCD diagnostic criteria. In this context, this review aims to familiarize dentists with OCD behavioral characteristics that were reported in dental clinics, which could be helpful to complete dental treatment sessions of patients with OCD successfully.

## 2. Materials and Methods

### 2.1. Protocol and Registration

The protocol of this review was performed in accordance with the guidelines of the Preferred Reporting Items for Systematic Reviews and Meta-Analyses (PRISMA) [4]. Registration of the protocol was done in International Prospective Register of Systematic Reviews (PROSPERO) platform with the Centre for Reviews and Dissemination at the University of York under code CRD42020212371.

### 2.2. Review Question

The focus question in this review was, “what are behavioral characteristics of individuals with OCD in dental clinics?”

### 2.3. Search Strategy

A structure online database search was conducted independently by the two reviewers the till the 24th of December 2020. Searches were performed on six different electronic databases: PubMed, Scopus, Web of science (WoS), Latin American & Caribbean Health Sciences Literature (LILACS), Cochrane Library, and PsycINFO. The organization and duplicated articles exclusion were carried out by EndNote online (Thomson Reuters, Philadelphia, USA). For each database, numerous words' combinations were done including “Obsessive compulsive disorder” AND “Dental” OR “Dentistry” OR “Periodontal” OR “Periodontitis” OR “Gingiva” OR “Orthodontic” OR “Dental implant” OR “Maxillofacial” OR “Teeth” OR “Tooth” OR “Tooth Extraction” OR “Pediatric dentistry” OR “Dental prosthesis” OR “Denture” OR “Bleaching” OR “Caries” OR “Amalgam” OR “Composite” OR “Oral lesion” OR “Tongue” OR “Edentulous” OR “Endodontic” OR “Pulp.”

### 2.4. Eligibility Criteria

Articles of this review were chosen based on the PICOS elements (Table 1), where population (P) = dental patients diagnosed with OCD, intervention (I) = different dental procedures, comparison (C) = dental patients free from OCD, outcome (O) = clinical difficulties during dental treatment sessions, and study design (S) = published case reports, case series, cohort, cross-sectional, case-control, or randomized clinical studies. Only articles that were published in English language were considered.

### 2.5. Study Selection

The study screening and selection process was carried out in two separate stages. At the first stage, the retrieved articles after the preliminary search of databases were evaluated according to their titles and abstracts by the two reviewers independently. The initial stage was followed by a second stage at which each identified study was reviewed in accordance with the inclusion/exclusion criteria by both reviewers, where each author constructed an independent list of selected articles. The lists were matched, revised, and compared. Duplicated studies were excluded. Contrarieties were finalized by discussion. The final list of the selected articles was checked independently for comprehensiveness and validity.

### 2.6. Data Extraction

The extracted and incorporated articles were piloted on standardized tables for data collection. Articles were screened and data from each article was extracted and tabulated in relation to demographic data such as author names and publication year and country. In addition, study design, sample size, characteristics of the participants, reported OCD behavior (as identified by NIMH), method of behavior assessment, acceptance of dental treatment, dental chief complaint, dental interventions, and major study findings were also collected in standard tables independently by the two reviewers.

### 2.7. Risk of Bias (Quality) Assessment

The quality of each included article was assessed independently by each author using the Joanna Briggs Institute's (JBI) Critical Appraisal Checklist for each type of the included studies [5]. Each article was assessed by multiple questions and the reviewer selected one answer for each question from “yes,” “unclear,” “no,” or “not applicable.” Articles were evaluated based on the following criteria: (LOW risk of bias) studies with more than 70% “yes” score; (MODERATE risk of bias) studies with 50% to 69% “yes” score; and (HIGH risk of bias) studies with less than 49% “yes” score. As recommended by the JBI reviewers' manual, all decisions regarding the scoring system and cut-off points were approved by all reviewers before the start of the critical appraisal process.

### 2.8. Syntheses of Results

The collected data from the included studies was qualitatively assessed for results synthesis. There was not enough homogeneity between collected articles, particularly in study design, and their outcomes. Henceforth, outcomes of these studies were explained in a narrative approach.

## 3. Results

### 3.1. Study Selection

**Figure 1:**
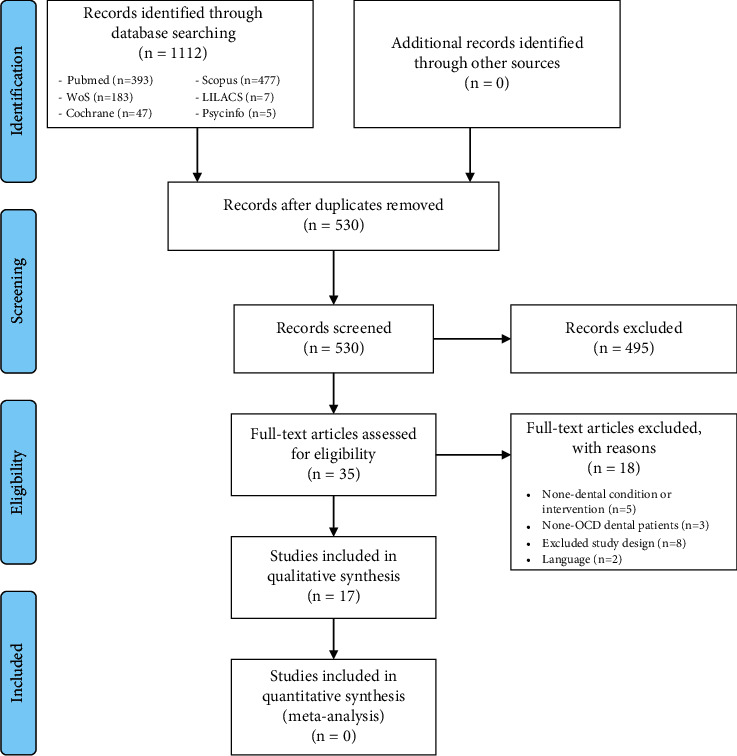
PRISMA flow diagram.

At the end of the conducted searches, a total of 1112 publications were retrieved. By removal of the repetitions, the number downgraded to 530 articles. In the preliminary stage, titles and abstracts of the 530 studies were evaluated and 35 studies remained eligible for full assessment. At the successional stage and after correlating the 35 studies with our mentioned inclusion criteria, only 17 studies met the requirements [6–22].  Table 2 defines the excluded 18 articles along with the exclusion reasons. Articles were mainly excluded due to one of the following reasons: 5 studies showed medical interventions without dental contribution [23–27], 3 studies reported dental patients with psychological disorder other than OCD [28–30], 8 articles were excluded because of their study design [31–38], and 2 articles were written in language other than English [39, 40]. In Figure 1, the PRISMA chart summarizes the study selection methodology.

### 3.2. Characteristics of Included Studies

Table 3 describes features of each included study in this review. All studies were conducted between 1991 and 2020. Studies were conducted in USA (5 articles), India (2 articles), Brazil (2 articles), Japan (2 articles), Spain (1 article), Korea (1 article), Canada/New Zealand (1 article), Greece (1 article), China (1 article), and Canada (1 article). Of the 17 included studies [6–22], 8 studies were case reports [6–13], while 5 studies were cross-sectional studies [14–18], 1 study was longitudinal cohort study [19], and 3 studies were case-control studies [20–22].

### 3.3. Results of the Individual Studies

As shown in Table 4, out of the 8 case reports, 4 patients were males, and 4 patients were females with age range from 10 to 63 years. Multiple dental symptoms were documented in these 8 case reports including tooth pain, dental caries, agenesis of lateral incisor, root fragment, impacted wisdom, missing teeth, complex vocal tics, severe lip biting, tooth wear, prolonged tooth brushing, gingival bleeding, painful palatal lesion, halitosis, and bruxism. All patients accepted the dental treatment except for one patient, where dental treatment was conducted after taking parent consent [7]. All cases (except two cases [7, 13]) reported at least one obsessions or compulsions symptom according to the NIMH. Fear of germs and contamination was reported in the works of Ahuja et al. [6], Chandna et al. [8], Michael [11], and Vieira et al. [12]. Unwanted forbidden or taboo thoughts involving sex, religion, or harm were not reported. Aggressive thoughts toward others or self were reported in the works of Fontenelle and Leite [9] and Herren and Lindroth [10]. Having things symmetric in perfect order was reported in the works of Ahuja et al. [6], Chandna et al. [8], and Herren and Lindroth [10]. Excessive cleaning or handwashing was reported in the work of Michael [11]. Ordering and arranging things in a particular way, repeatedly checking things, and compulsive counting were reported in the works of Ahuja et al. [6] and Chandna et al. [8]. All patients had OCD with no other psychological disorder except for one patient who was schizophrenic [7].  Table 5 shows the study population and inclusion criteria for all selected clinical investigations. For the 5 cross-sectional studies, dental specialties were maxillofacial surgery [14, 16, 18], psychosomatic dentistry [15], and oral medicine [17]. For the longitudinal cohort study, dental specialty was general [19]. For the 3 case-control studies, dental specialties were oral medicine and temporomandibular disorders (TMD) [20–22]. Multiple behavioral assessment methods for OCD were carried out in the selected clinical studies including Florida Obsessive-Compulsive Inventory (FOCI) in the work of Haberle et al. [14]; the Symptom Checklist-90-Revised (SCL-90-R) in the works of Kim et al. [20], Phillips et al. [16], Velly et al. [21], and Liu et al. [18]; Diagnostic Interview Schedule (DIS) in the work of Locker et al. [19]; Diagnostic and Statistical Manual of Mental Disorders, Fifth Edition (DSM-5) in the works of Miura et al. [15] and Umemura et al. [17]; and the Crown Crisp Experimental Index (CCEI) in the work of Zach and Andreasen [22].

### 3.4. Studies' Risk of Bias (Quality) Assessment

Assessment of the included articles' quality is demonstrated in Table 6. After the evaluation using JBI's Critical Appraisal Checklist for case reports, cross-sectional, cohort, and case-control studies, all articles received an acceptable quality appraisal to be included in the current review. Out of the eight evaluated case reports, seven showed low risk of bias, while one case report [10] showed moderate risk of bias. The overall scores for case reports were as follows: 75% for studies of Ahuja et al. [6], Castellanos-Cosano et al. [7], and Chandna et al. [8]; 87.5% for studies of Fontenelle and Leite [9], Michael [11], Vieira et al. [12], and Oulis et al. [13]; and 62.5% for the study of Herren and Lindroth [10]. Of the five analyzed cross-sectional studies, four articles were scored as low risk of bias, while one article [17] showed moderate risk of bias. The overall scores for cross-sectional studies were as follows: 75% for studies of Haberle et al. [14], Miura et al. [15], Phillips et al. [16], and Liu et al. [18] and 62.5% for Umemura et al.'s study [17]. The only evaluated cohort study was Locker et al.'s [19] which showed low risk of bias with appraisal score of 75%. Out of the three assessed case-control studies, two exhibited low risk of bias, while one article [22] showed moderate risk of bias. The overall scores for case-control studies were as follows: 80% for Kim et al.'s study [20], 70% for Velly et al.'s study [21], and 60% for the study of Zach and Andreasen [22].

### 3.5. Syntheses of Results

The outcomes of all included clinical investigations were collected and tabulated (Table 5). For instance, clinical studies varied in their design including 5 cross-sectional studies, 1 longitudinal cohort study, and 3 case-control studies. Case reports were mainly a qualitative description of the reported cases. In addition, qualitative assessment of the included clinical studies revealed separate outcomes. One study suggested that the behavior of patients diagnosed with OCD could be improved following orthognathic surgeries [14]. Another study showed that trauma history in patients with TMD may have psychological impact [20]. Studies also found a direct proportional correlation between dental anxiety and psychological disorders such as OCD [18, 19]. Psychological disorders in general were considered to be linked with different dental-related problems including atypical odontalgia [15], dentofacial correctness seeking [16], pain [17, 21], and temporomandibular joint (TMJ) problems [22]. Therefore, findings of these studies were discussed in comprehensive manner.

## 4. Discussion

Several studies have documented the impact of psychologic disorders and patient's mental health status on the oral health and dental practice. In the present review, we reported data from published literature on OCD-related psychological behaviors and their impact on dental treatment procedures and session planning. Obsessions or compulsions symptoms were documented in case reports of dental patients with OCD such as fear of germs and contamination, aggressive thoughts toward others or self, having things symmetric in perfect order, excessive cleaning or handwashing, ordering, arranging things in a particular way, repeatedly checking things, and compulsive counting were reported. Large clinical studies also reported OCD-related behaviors in dental patients that were assessed by multiple behavioral assessment methods including FOCI [41], SCL-90-R [42], DIS [43], DSM-5 [44], and CCEI [45].

Assessing the behavior of OCD dental patients was conducted in this review. In the eight selected case reports, dental patients with OCD showed different OCD-related obsessions or compulsives that resulted in difficulties or modifications of their dental procedures. Ahuja et al. [6] and Chandna et al. [8] stated that their patients could get agitated easily, if they were not relieved frequently through the treatment sessions that everything around them such as dental tools and dental chair is cleaned and disinfected. Sometimes things were cleaned in front of them to reassure them. They also used to count in a specific pattern during the treatment. Castellanos-Cosano et al. [7] showed that there was no contraindication in placing a dental implant in a patient with OCD after consulting the patient's psychologist. They also pointed that their patient may refuse the dental treatment but, with the guardian consent, treatment can be conducted safely. Fontenelle and Leite [9] demonstrated that their patient developed oral self-mutilations resistant to medications. However, these symptoms subsided after fabricating an ordinary mouth guard. The included case reports also argued that OCD-related thoughts can lead to serious dental conditions. Herren and Lindroth [10] revealed that their patient was locked up in the idea of tapping on her teeth in a specific manner before carrying out her daily activities, which results in multiple teeth wear. The dentist in this case preferred to postpone her dental treatment to allow drugs for OCD and behavioral therapy to take place first. Moreover, Michael [11] declared that his case was caged in the idea of teeth brushing that took a very long time (up to four hours every night), which affected her day schedule. After adopting exposure and response protocol in treatment of OCD, the patient eventually started to take over her inner thoughts of prolonged teeth brushing. Vieira et al. [12] showed that the patient consumed unhealthy diet for years due to inner fears of insecticides in vegetables. He also had similar fears toward chemicals in toothpastes, which was reflected on his tooth brushing habits. All these behaviors ended with scurvy presented with severe gingival bleeding and palatal lesions. After modulating his diet and introducing vitamin C supplement, symptoms started to vanish. Oulis et al. [13] presented an interesting case of a woman with a 40-year history of severe OCD managed by fluoxetine or escitalopram. She developed bruxism because of these medications. However, symptoms of bruxism subsided after adding aripiprazole to her regimen.

The selected clinical studies also reported correlation between OCD behaviors and dental complains or symptoms. Haberle et al.'s [14] study identified OCD as one of the common comorbid symptoms noticed among patients undergoing orthognathic surgeries. The OCD symptoms were markedly diminished postoperatively as indicated by reduced index of FOCI. This made the authors suggest that jaw deformities may induce more psychological concerns or even obsessions regarding the facial appearance. Out of the 383 patients with atypical odontalgia in Miura et al.'s work [15], 177 showed comorbid psychiatric disorders including OCD, which was observed in 4 patients (1%). Overall, this study suggested that psychological disorders such as OCD in patients with atypical odontalgia might trigger pain emotional response. In Phillips et al.'s study [16], 194 patients, who were going to have orthognathic surgeries, completed the SCL-90-R to assess their psychological distress. Around 15% of study population had clinical elevation toward psychological disorders such as OCD. This study noted that people with OCD are more likely to seek for facial deformities correctness, which might come from their inner thoughts of perfectionism. In Umemura et al.'s study [17], 1202 patients with psychiatric illness were evaluated based on the DSM-5. 0.3% of the patients (4 patients) were diagnosed with OCD. One patient with OCD showed oral manifestation such as oral dysesthesia. This study suggested that, beside physiological causes, psychiatric disorders augment orofacial pain. In Liu et al.'s study [18], the preoperative anxiety along with postoperative satisfaction was assessed in 92 patients undergoing anterior dental implant surgeries. In this study, evaluation of OCD symptoms by SCL-90-R revealed that SCL-90-R scores were not significant between study subjects and norm of Chinese. Locker et al. [19] assessed dental anxiety and psychological disorders, such as major depressive episode, dysthymia, generalized anxiety disorder, panic disorder, agoraphobia, social phobia, simple phobia, obsessive-compulsive disorder, conduct disorder, cannabis, and alcohol dependence among 805 subjects using the Dental Anxiety Scale (DAS) [46] and the DIS, respectively. The prevalence of OCD was 4.1% with no significant difference between males and females. In this study, 4% of the nonanxious dental patients had OCD, while OCD was reported in 3.2% and 8.3% of the moderately and severely anxious dental patients, respectively. After correlating, it was found that, within the selected population, the assessed psychological disorders play a significant role in the maintenance of dental anxiety. In Kim et al.'s study [20], the SCL-90-R was employed for the assessment of the psychological characteristics, including OCD, in patients with TMD. Collectively, this study concluded that trauma history may induce additional significant subjective, objective, and psychological impairments among patients diagnosed with TMD. Velly et al. [21] correlated masticatory myofascial pain (MFP) and psychological factors (assessed by SCL-90-R) in 83 chronic MFP cases and 100 controls. 55 MFP and 74 controls showed symptoms of OCD behaviors. Consequently, the authors proposed that MFP and psychological symptoms might be bidirectionality correlated. Zach and Andreasen [22] evaluated the psychological features of 98 females with TMD symptoms using the CCEI against 98 other control females. The mean OCD profile scores between patients with TMD and control were 5.32 (±3.15) and 4.89 (±2.82), respectively, whereas the difference between the two means was not statistically significant (*p*=0.3358). Although this study did not report direct correlation between OCD symptoms and TMD, it showed that OCD may have impact on dental health after excluding nonorganic reasons.

To the best of our knowledge, this is the first review that comprehensively reports the published behavioral symptoms of patients with OCD in dental clinics to evaluate the impact of these psychological symptoms on dental procedures and sessions planning. Another strength point in this review is that articles selection procedure was adherent to the PRISMA guidelines along with prior registration of the protocol with the PROSPERO database, which indicates greater quality of the reporting with minimal bias risks. We were not able to conduct meta-analysis in the present review due to the heterogeneity among the included studies. The included reports qualitatively described the cases with no reported quantitative assessment. In the clinical studies, assessment of the OCD behavioral related symptoms was carried out using multiple procedures. In addition, several dental specialties were involved, which were associated with variable dental sign and complaints. Outcomes and conclusion of these clinical studies were correlated with psychiatric and psychological disorders in general, which could not be applied on patients diagnosed with OCD specifically due to their low number.

## 5. Conclusion

According to the selected articles, symptoms of OCD could be reported in dental clinics. OCD-related obsessions or compulsive behaviors might produce some difficulties during dental procedures but do not markedly affect the dental treatment. Moreover, OCD is not contraindicated in dental treatments including surgeries such as orthognathic surgeries or placement of dental implants. Like other psychological disorders, OCD might add an extra dental anxiety burden and influence the patient's satisfaction. Dentists should consider flexibility during treating patients with OCD.

## Figures and Tables

**Table 1:** PICOS question of the review.

PICOS	Inclusion criteria	Exclusion criteria
Population	Patients having dental or oral condition and diagnosed with OCD.	Patients with or without psychological disorder other than OCD.

Intervention	All dental procedures including preventative, conservative, or maxillofacial surgeries.	Medical interventions without dental contribution.

Comparison	Mental and psychologically normal dental patients.	Dental patients with psychiatric disorder other than OCD.

Outcome	Practicing difficulties associated with patients with OCD in dental clinics.	Clinical practicing complications related to people with OCD in nondental clinics.

Study design	Published case reports, case series, cohort, cross-sectional, case-control, or randomized clinical studies.	Conference abstract, editorial correspondence, book chapters, studies not involving human subjects, or review articles

**Table 2:** Excluded articles and causes of exclusion.

No.	Author	Title	Causes of exclusion
1	Arasteh et al. [23]	Frequency of obsessive-compulsive symptoms and related factors in medical and dental students of kurdistan university of medical sciences, 2018	Assessment of OCD was carried out in medical and dental students. Dental patients were not involved in this study.

2	Budman and Sarcevic [24]	An unusual case of motor and vocal tics with obsessive-compulsive symptoms in a young adult with Behçet's disease	A case report that describes nondental patient with Behcet's disease who showed motor and vocal tics with OCD.

3	Cassin et al. [25]	Quality of life in treatment-seeking patients with obsessive-compulsive disorder with and without major depressive disorder	A study that compares the quality of life of a patient with OCD in relation to depression comorbidity. Dental patients were not involved in this study.

4	Cockburn et al. [31]	Oral health impacts of medications used to treat mental illness	The study design is a review article.

5	de Jongh [39]	Mental problems in the dental practice: a compulsive disorder	The article was written in Dutch.

6	De Stefano et al. [32]	Fear and anxiety managing methods during dental treatments: a systematic review of recent data	The study design is a review article.

7	Dougall and Fiske [33]	Access to special care dentistry, part 6. Special care dentistry services for young people	The study design is a review article.

8	Doukhan et al. [26]	A case of bleach addiction associated with severe obsessive-compulsive disorder	A case report that describes a patient having OCD with bleach use addiction (not related to dental use or teeth bleaching).

9	Elmgreen and Danielsen [40]	OCD and orofacial dyskinesia caused by a rare basal ganglia disorder	The article was written in Danish.

10	Friedlander and Eth [34]	Dental management considerations in children with obsessive-compulsive disorder	The study design is a review article.

11	Friedlander and Serafetinides [35]	Dental management of the patient with obsessive‐compulsive disorder	The study design is a review article.

12	Friedlander and Cummings [36]	Dental treatment of patients with Gilles de la Tourette's syndrome	The study design is a review article.

13	Hollander et al. [28]	A placebo controlled crossover trial of liquid fluoxetine on repetitive behaviors in childhood and adolescent autism	The article describes the effects of fluoxetine as a selective serotonin reuptake inhibitor liquid on the repetitive behaviors in 45 child or adolescent patients with autism spectrum disorders (ASDs). OCD behaviors were not reported in this study.

14	Kayhan et al. [27]	Obsessive-compulsive disorder concurrent with Melkersson-Rosenthal Syndrome: A case report	A case report that describes a patient with OCD and Melkersson-Rosenthal Syndrome (MRS). The patient was admitted to psychiatry department without reporting dental-related symptoms.

15	Keim [37]	The most difficult cases	The article is an editor's corner.

16	Moore and Hersh [38]	Common medications prescribed for adolescent dental patients	The study design is a review article.

17	Paterson and Watson [29]	Case report: prolonged match chewing: an unusual case of tooth wear	A report that describes a case of sand eating habit that caused abrasive tooth wear. The patient was not diagnosed with OCD.

18	Rahman et al. [30]	Oral health status of patients with psychiatric problem	A cross-sectional study that examined the oral health status of 75 psychiatric patients. The major reported mental illness was schizophrenia. OCD was not reported in these patients.

**Table 3:** Characteristics of the included studies.

No.	Author	Title	Country	Year	Study design
1	Ahuja et al. [6]	Juvenile obsessive compulsive disorder in a paediatric dentistry setup	India	2015	Case report

2	Castellanos-Cosano et al. [7]	Dental implants placement in paranoid squizofrenic patient with obsessive-compulsive disorder: a case report	Spain	2017	Case report

3	Chandna et al. [8]	Obsessive compulsive disorder in dental setting	India	2014	Case report

4	Fontenelle and Leite [9]	Treatment-resistant self-mutilation, tics, and obsessive-compulsive disorder in neuroacanthocytosis: a mouth guard as a therapeutic approach	Brazil	2008	Case report

5	Herren and Lindroth [10]	Obsessive compulsive disorder: a case report	USA	2001	Case report

6	Michael [11]	Cognitive-behavioral treatment of obsessive-compulsive disorder: a case of prolonged tooth brushing	USA	2006	Case report

7	Vieira et al. [12]	Scurvy induced by obsessive-compulsive disorder	Brazil	2009	Case report

8	Oulis et al. [13]	Low-dose aripiprazole in the treatment of SSRI-induced bruxism	Greece	2012	Case report

9	Haberle et al. [14]	Body image disturbance and obsessive-compulsive disorder symptoms improve after orthognathic surgery	USA	2020	Cross-sectional

10	Miura et al. [15]	Psychiatric comorbidities in patients with atypical odontalgia	Japan	2018	Cross-sectional

11	Phillips et al. [16]	Dentofacial disharmony: psychological status of patients seeking treatment consultation	USA	1998	Cross-sectional

12	Umemura et al. [17]	Oral medicine psychiatric liaison clinic: study of 1202 patients attending over an 18-year period	Japan	2019	Cross-sectional

13	Liu et al. [18]	Preoperative anxiety decreases the postoperative satisfaction in anterior dental implant surgery	China	2016	Cross-sectional

14	Lockeret al. [19]	Psychological disorders and dental anxiety in a young adult population	Canada/New Zealand	2001	Longitudinal cohort study

15	Kim et al. [20]	Clinical and psychological characteristics of TMD patients with trauma history	Korea	2010	Case-control study

16	Vellyet al. [21]	Contributing factors to chronic myofascial pain: A case-control study	Canada	2003	Case-control study

17	Zach and Andreasen [22]	Evaluation of the psychological profiles of patients with signs and symptoms of temporomandibular disorders	USA	1991	Case-control study

**Table 4:** Characteristics of included case reports in relation to OCD behavior in dental clinics.

No.	Author	Patient's gender (age in years)	Dental symptoms	Accepted dental treatment?	Reported obsessions^*∗*^	Reported compulsions^*∗∗*^	Other psychological disorders	Study outcome
1	Ahuja et al. [6]	Male (10)	Tooth pain and dental caries	Yes	a and d	b, c, and d	Not reported	Dental treatment of a patient with OCD may take an awkward path due to the patient's demands but considering shortening the treatment session and being flexible according to patient's behavior may be beneficial in avoiding repetitive routines in every step.

2	Castellanos-Cosano et al. [7]	Male (33)	Agenesis of lateral incisor, root fragment, impacted wisdom, and missing teeth	No (parent consent was taken)	Not reported	Not reported	Schizophrenic	Implants can be loaded successfully in patients with OCD after consultation with the patient's psychiatric.

3	Chandna et al. [8]	Female (11)	Tooth pain and dental caries	Yes	a and d	b, c, and d	Not reported	OCD diagnosis can be made in dental clinic by correlating patient's history and behavior during treatment sessions with OCD criteria.

4	Fontenelle and Leite [9]	Male (32)	Complex vocal tics and severe lip biting	Yes	C	Not reported	Not reported	Self-mutilation resistant to medications can be treated with a simple mouth guard.

5	Herren and Lindroth [10]	Female (30)	Tooth wear	Yes	c and d	Not reported	Not reported	Elective restorative treatment could be postponed allowing for medications and behavioral therapy. Dental treatment was completed after few months.

6	Michael [11]	Female (11)	Prolonged tooth brushing up to 4 hours	Yes	A	a	Not reported	Exposure and response procedure is one of the most efficient protocols in treatment of OCD children.

7	Vieira et al. [12]	Male (61)	Gingival bleeding, painful palatal lesion, and halitosis	Yes	A	Not reported	Not reported	Bizarre OCD behavior of the patient and unhealthy diet lead him to scurvy.

8	Oulis et al. [13]	Female (63)	Involuntary jaw movements, teeth clenching, and severe teeth pain.	Not applicable	Not reported	Not reported	Not reported	Bruxism induced by OCD medications was treated by aripiprazole.

^*∗*^Obsessions: a: fear of germs and contamination; b: unwanted forbidden or taboo thoughts involving sex, religion, or harm; c: aggressive thoughts toward others or self; d: having things symmetric in perfect order. ^*∗∗*^Compulsions: a: excessive cleaning or handwashing; b: ordering and arranging things in a particular way; c: repeatedly checking things; d: compulsive counting.

**Table 5:** Characteristics of included clinical studies in relation to OCD behavior in dental clinics.

No.	Author	Population	Dental specialty	Study design	Inclusion criteria	Method of OCD behavior assessment	Study outcome
1	Haberle et al. [14]	Total 49 patients (21 females and 28 males). Age between 13 and 56 years.	Maxillofacial surgery	Cross-sectional (questionnaire)	Patients with a maxillomandibular deformity who require surgical intervention. No previous maxillomandibular surgery. No TMJ joint disorder. No craniofacial deformity.	Florida Obsessive-Compulsive Inventory (FOCI)	OCD symptoms improved after orthognathic surgeries

2	Miura et al. [15]	Total 383 patients (58 males and 325 females). Age between 18 and 86 years.	Psychosomatic dentistry	Cross-sectional	Above 18 years of age. Tooth pain for no reason for more than 6 months. Pain following tooth extraction without underlying pathology.	The Diagnostic and Statistical Manual of Mental Disorders, Fifth Edition (DSM-5)	Psychiatric disorders are one of the reasons behind atypical odontalgia.

3	Phillips et al. [16]	Total 194 patients (72 males and 122 females). Age between 15 and 50 years.	Maxillofacial surgery	Cross-sectional	Age between 15 and 50 years. Anterior-posterior or vertical deformities. Require surgical treatment. Noncongenital deformity. No previous prosthesis.	The Symptom Checklist-90 (SCL-90)	Psychological disorders can be the reason for many people to seek dentofacial correctness.

4	Umemura et al. [17]	Total 1202 patients (210 males and 992 females). Age: 57.2 ± 15.0 years.	Oral medicine	Cross-sectional	Dental patients with prolonged oral pain without organic cause.	The Diagnostic and Statistical Manual of Mental Disorders, Fifth Edition (DSM-5)	Psychiatric disorders can be the reason for oral or dental pain after exclusion of organic reasons.

5	Liu et al. [18]	Total 92 patients (55 males and 37 females). Age above 18 years.	Maxillofacial surgery	Cross-sectional	Above 18 years of age. Free from systematic diseases and metastatic lesions. No history of alcohol abuse. No history of immunity medications or beta blockers for the last six months.	Symptom Checklist 90 (SCL-90)	Anxious patients and patients with psychological disorders are more likely to feel pain during implant placement and are more expected to show dissatisfaction with results.

6	Locker et al. [19]	Total 805 patients (413 males and 392 females). Age between 3 and 26 years.	General	Longitudinal cohort study	Children born in New Zealand in Queen Mary Hospital. Period between April 1972 and March 1973. Their mothers lived in Dunedin Metropolitan Area.	Diagnostic Interview Schedule (DIS)	Dental anxiety has a direct proportional relation with psychological disorders.
7	Kim et al. [20]	Total 34 patients (10 males and 24 females). Total 340 control (100 males and 240 females). Age (males: 28.4 ± 6.6 years and females: 35.9 ± 14.0 years).	Oral medicine	Case-control	TMD patients following physical trauma. Control: TMD without physical trauma.	The Symptom Checklist-90-Revised (SCL-90-R)	TMD patients with trauma history displayed more severe subjective, objective, and psychological dysfunction than those without trauma history.

8	Velly et al. [21]	Total 83 patients (16 males and 67 females). Total 100 control (36 males and 64 females). Age between 18 and 60 years.	TMD	Case-control	Age between 18 and 60 years. No pregnancy. Reading and speaking English or French. Nondental pain.	The Symptom Checklist-90-Revised (SCL-90-R) questionnaire.	Psychological disorders may be a contributing factor to myofascial pain.

9	Zach and Andreasen [22]	Total 98 female patients. Total 98 female control. Age above 18 years.	TMD	Case-control	Females above 18 years of age	The Crown Crisp Experimental Index (CCEI).	Psychological factors may play a role in etiology of TMJ problems.

**Table 6:** Response to JBI's Critical Appraisal Checklist for each type of the included studies (assessment of risk of bias).

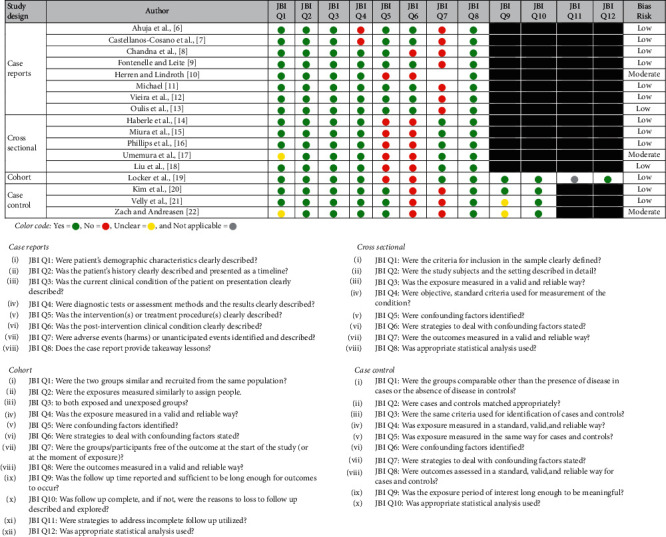

## Data Availability

The data used to support the findings of this study are included within the article.
